# Supplementary material for: Guiding the Refinement of Biochemical Knowledgebases with Ensembles of Metabolic Networks and Machine Learning
Source: Cell Syst. 2020 Jan 22;10(1):109–119.e3. doi: 10.1016/j.cels.2019.11.006 (PMC6975163; doi:10.1016/j.cels.2019.11.006)
Supplement: Document S1. Figures S1 and S2 [file mmc1.pdf]

Cell Systems, Volume *10*

Supplemental Information

Guiding the Refinement of Biochemical Knowledgebases with Ensembles of Metabolic Networks and Machine Learning

Gregory L. Medlock and Jason A. Papin

Supplemental Figures

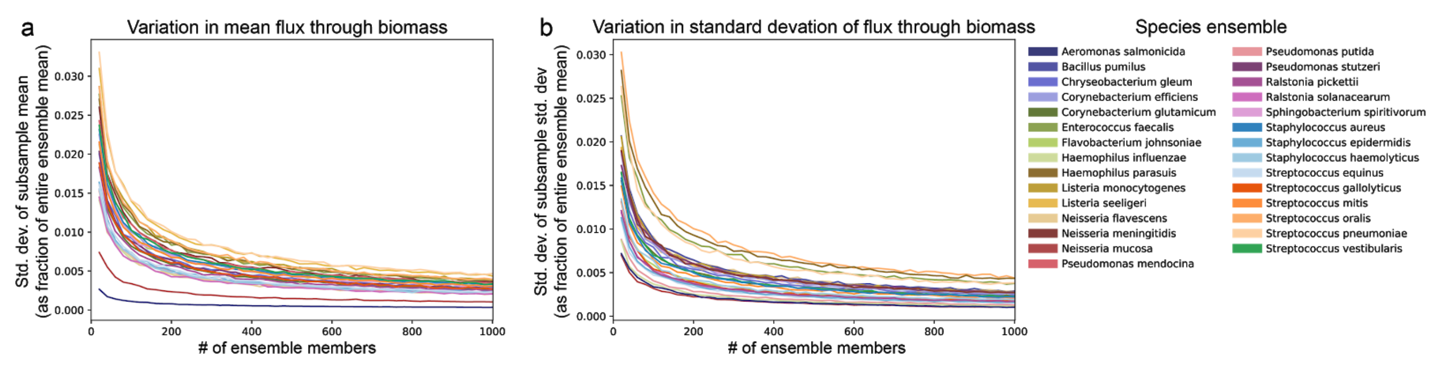

Figure S1. Subsampled ensemble behavior for predictions of biomass production, related to **Figure 2**. We simulated biomass production in a rich medium across the entire ensemble and subsampled these results at varying ensemble sizes. **a)** Standard deviation of the mean flux through biomass from each subsample and **b)** standard deviation of the standard deviation of flux through biomass in each subsample. For both quantities (variance of the mean of each subsample and variance of the variance of each subsample), simulations plateau before inclusion of all 1000 ensemble members. Values on the y axis are normalized by dividing by the mean flux through biomass for the entire ensemble.

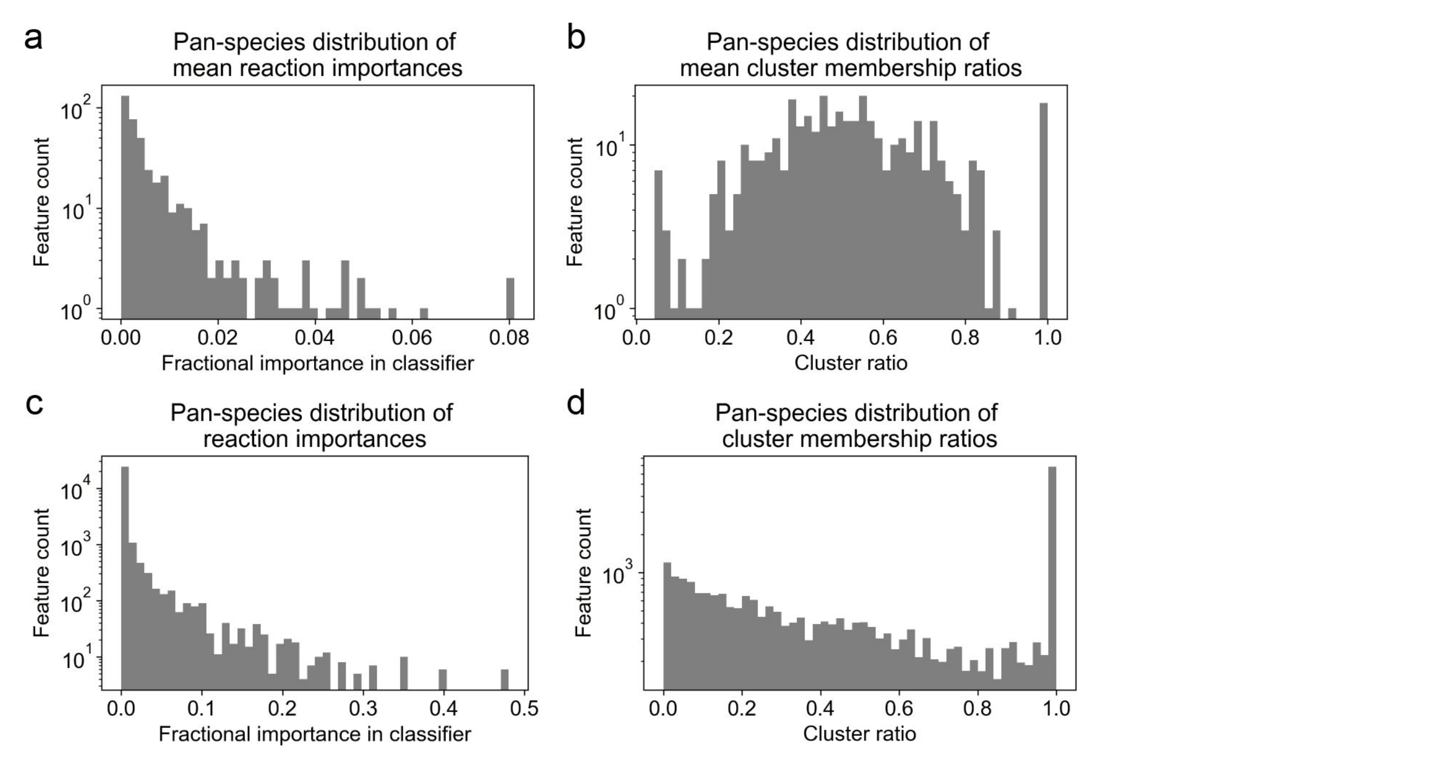

Figure S2. Distribution of fractional importances and cluster ratios, related to **Figure 4**. **a)** Distribution of mean fractional importances for reactions gap-filled in at least 5 ensembles. Identical to **Figure 4b** other than filtering step. **b)** Distribution of mean cluster ratios for reactions gap-filled in at least 5 ensembles. Identical to **Figure 4c** other than filtering step. **c)** Distribution of reaction importances across all species. Identical to **Figure 4b** except the mean is not taken across all species; the distribution includes values for individual reactions instead of a mean (e.g., a reaction occurring in 7 species has 7 values that are part of the distribution, rather than a single mean as in **Figure 4b**). **d)** Distribution of cluster ratios across all species. As in **c**, the mean is not taken and individual values are included.
